# Anterior segment migration of intravitreal dexamethasone implant in a patient with scleral fixation intraocular lens implant: a case report

**DOI:** 10.1093/jscr/rjae121

**Published:** 2024-03-08

**Authors:** Hani B ALBalawi, Naif M Alali, Abdullah H Altemani, Moustafa S Magliyah, Khaled S ASharari, Maram S ALRubayyi, Faris Hashem, Saad H Alenezi

**Affiliations:** Division of Ophthalmology, Department of Surgery, Faculty of Medicine, University of Tabuk, Tabuk 71491, Saudi Arabia; Vitreoretinal Division, King Khaled Eye Specialist Hospital, Riyadh 12329, Saudi Arabia; Division of Ophthalmology, Department of Surgery, Faculty of Medicine, University of Tabuk, Tabuk 71491, Saudi Arabia; Department of Family and Community Medicine, Faculty of Medicine, University of Tabuk, Tabuk 71491, Saudi Arabia; Vitreoretinal Division, King Khaled Eye Specialist Hospital, Riyadh 12329, Saudi Arabia; Ophthalmology Department, Prince Mohammed Medical City, Aljouf 72345, Saudi Arabia; Vitreoretinal Division, King Khaled Eye Specialist Hospital, Riyadh 12329, Saudi Arabia; Ophthalmology Department, Prince Mohammed Medical City, Aljouf 72345, Saudi Arabia; Radiology Department, Security Force Hospital, Riyadh 11564, Saudi Arabia; Division of Ophthalmology, Department of Surgery, Faculty of Medicine, University of Tabuk, Tabuk 71491, Saudi Arabia; Department of Ophthalmology, Majmaah University, Majmaah 15341, Saudi Arabia

**Keywords:** Ozurdex implant migration, conservative management, dexamethasone, macular edema, Ozurdex complications

## Abstract

Corticosteroids are crucial for treating inflammatory ocular conditions. The development of dexamethasone revolutionized targeted ocular therapy. Ozurdex, a dexamethasone implant, effectively treats various eye conditions but carries risks such as implant migration. This is a case of anterior segment migration of intravitreal dexamethasone implant, Ozurdex, in a patient with scleral fixation intraocular lens implant in whom conservative management with supine positioning and pharmacologic pupil dilation can help retain the implant back in the vitreous. Patients at high risk of Ozurdex migration should avoid its use. Educate patients on the risk of implant migration and signs of migration to present immediately to an ophthalmology emergency department to avoid corneal damage. It is essential to identify high-risk patients before considering Ozurdex migration. In some cases, conservative management can be initiated while preparing for surgical removal.

## Introduction

Corticosteroids have anti-inflammatory and anti-angiogenic properties that make them an ideal therapeutic option for a variety of posterior segment diseases [1, 2], as many highly prevalent ocular diseases are initiated by inflammation and angiogenesis factors [3]. In the 1950s, the use of corticosteroids in treating ocular diseases started when ophthalmologists used corticosteroids to treat uveitis [4–6]. The ocular inflammation had been treated previously by elevating body temperature to induce endogenous corticosteroid production [7]. Dexamethasone was first made in 1957 and was approved for medical use in 1961 [8, 9]. Ozurdex is a biodegradable, sustained-release intravitreal implant containing 0.7-mg of preservative-free Dexamethasone of preservative-free Dexamethasone [10]. In 2009, it was approved by the US Food and Drug Administration to treat macular edema following branch retinal vein occlusion (BRVO) and central retinal vein occlusion (CRVO), and also, for treating non-infectious uveitis affecting the posterior segment of the eye. However, it is contraindicated in ocular or periocular infections, advanced glaucoma, non-intact posterior lens capsules, and hypersensitivity [11]. Reported side effects include intravitreal injection-related side effects like endophthalmitis [12] and steroid-related side effects like posterior subcapsular cataracts, increased intraocular pressure, and others [13]. Patients whose posterior capsule of the lens is absent or defective, have zonular damage, or have a history of vitrectomy are associated with migration of dexamethasone intravitreal implants into the anterior chamber [14]. This report describes a case in a hospital, of a pseudophakic patient with scleral fixation IOL presented 2 weeks after the Ozurdex procedure with anterior chamber migration of implant in which conservative measurement resolved the migration and avoidance of the surgical intervention for implant removal.

### Case report

**Figure 1:**
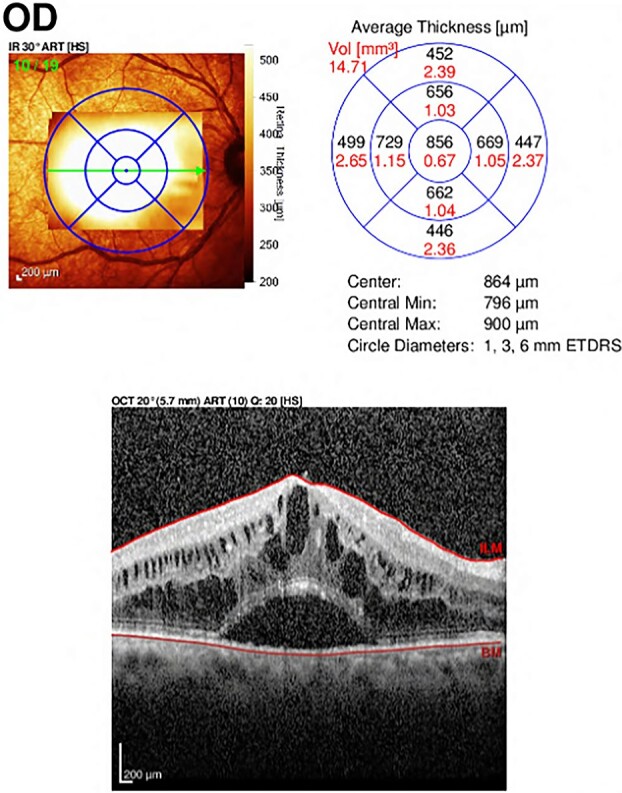
Optical Coherence Tomography (OCT) of the right eye showed central involvement macular edema with intra-retinal and sub-retinal fluid accumulation.

**Figure 2:**
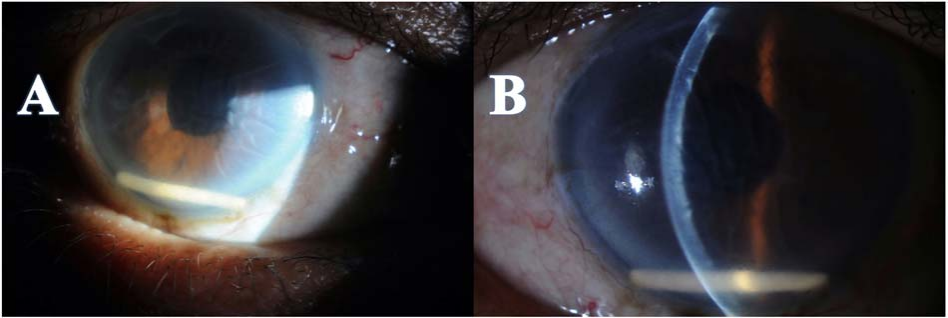
Anterior segment photo showed the Ozurdex implant migration to the anterior chamber with secondary corneal edema (A), with Descemet folds (B).

A 66-year-old male with a history of ‘phacoemulsification’ with posterior chamber ‘intraocular lenses (IOL) implantation5 years back’ in the right eye. Three years later, the patient returned with dislocated IOL in the anterior chamber due to trauma. The patient underwent IOL removal with anterior vitrectomy and scleral fixated IOL. Seven months postoperatively, the patient complained of blurry vision in the right eye. The patient was referred to the retina service, which showed the visual acuity in the right eye was 20/80, the left eye 20/30, and intraocular pressure (IOP) was 16 OU; slit lamp biomicroscopic examination showed the lid and conjunctiva were within normal limits bilaterally. The corneal exanimation shows normal and clear cornea in both eyes; the anterior chamber was deep and quiet bilaterally. The pupil was normal in size with no relative afferent pupillary defect. The scleral fixed IOL is in place OD, and PC-IOL is in place OS. A fundoscopic examination of both eyes indicates a flat retina, normal optic disc, and no vasculitis or retinitis in either eye. In the right eye, a macular edema with central involvement was detected. The patient was diagnosed with pseudophakic cystoid macular edema (Fig. 1) with a central macular thickness of 856 μm. The patient underwent an intravitreal dexamethasone implant (Ozurdex) in the right eye. Two weeks later, the patient presented to the emergency department complaining of painless decreased vision of the right eye with a whitish discoloration object seen behind the cornea. A slit-lamp examination of the right eye indicated mild conjunctival injection and moderate corneal edema. The Ozurdex implant was in the inferior aspect of the anterior chamber, and the IOL was in place with normal IOP. Slit-lamp photos were taken to show the Ozurdex implant in the anterior chamber (Fig. 2 A and B). B-scan ultrasonography indicated no implant in the posterior chamber. The patient was admitted for bed rest in the supine position, cycloplegic, and scheduled for Ozurdex implant removal with anterior chamber washout the next day.

**Figure 3:**
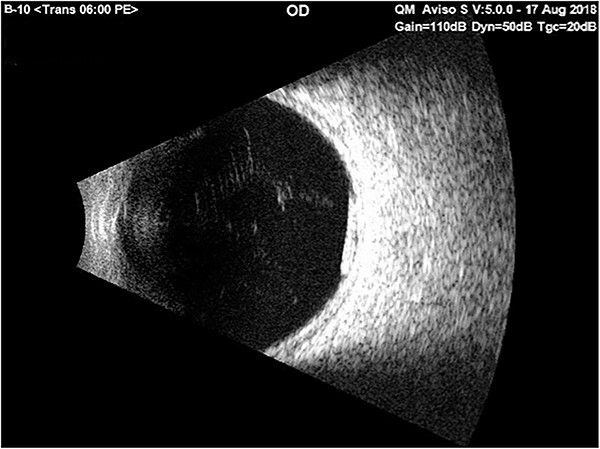
Ultrasound *b*-*scan* exam of the right eye showed a reflective small tube inferior and posterior to the equator on the 1st day of admission with only bed rest in the supine position and using cycloplegic drops.

Just before the planned implant removal surgery, the examination indicated no Ozurdex implant in the anterior chamber; the surgery was postponed for re-evaluation. *B*-*scan* ultrasonography revealed the Ozurdex implant was back in the vitreous and showed a highly reflective small tube inferior and posterior to the equator and freely mobile (Fig. 3). The patient was discharged with hypertonic saline and topical prednisolone for corneal edema. On follow-up 2 weeks later, the fundus examination showed that the implant was still in place at the vitreous.

## Discussion

The largest series of patients with Ozurdex implants with anterior chamber migration was reported by Khurana *et al.* They reviewed the risk factors, management, and complications of 15 patients who had prior pars plana vitrectomy, and 93% had no lens capsule. They reported that the average interval from Ozurdex implant injection to the detection of implant migration into the anterior chamber was 13 days. They also reported on two patients with a scleral-fixated posterior chamber IOL. Their first case was treated with surgical removal with no resolution of cornea edema, and the other case was treated with supine bed rest and corneal transplant [14]. Management options in literature for Ozurdex implant with anterior chamber migration include observation, supine positioning, YAG fragmentation, and surgical removal involving either forceps, aspiration of implant fragments, or repositioning into the posterior chamber [15].

Our patient had an increased risk for migration because he had undergone an anterior vitrectomy, and there was no capsule and a scleral fixated IOL, so it is better to avoid the use of Ozurdex in high-risk migration patients. However, conservative management with supine positioning and pharmacological pupil dilation can help in returning the migrated Ozurdex implant. Early surgical intervention can help avoid corneal decompensation. We advise that the patient be educated on the risk of implant migration and the signs of migration to present immediately to an ophthalmology emergency department to avoid corneal decompensation. Knowing the high risk of Ozurdex migration patients is critical in choosing candidates. Starting conservative management while preparing patients for surgical removal can help in some cases.

## Data Availability

All data generated or analysed during this study are included in this published article.
